# Chemical Characterization of the Marking Fluid of Breeding and Non-Breeding Male Cheetahs

**DOI:** 10.3390/ani12172284

**Published:** 2022-09-03

**Authors:** Alexia Tommasi, Andreas G. J. Tredoux, Jacek A. Koziel, Giulia Esposito

**Affiliations:** 1 Department of Animal Sciences, Stellenbosch University, Stellenbosch 7600, South Africa; 2 Department of Chemistry, Stellenbosch University, Stellenbosch 7600, South Africa; 3 USDA-ARS Conservation and Production Research Laboratory, Bushland, TX 79012, USA; 4 Department of Veterinary Science, University of Parma, 43126 Parma, Italy

**Keywords:** marking fluid, gas-chromatography mass-spectrometry, reproductive behavior, Felidae, *Acinonyx jubatus*, cheetahs, chemical communication, volatile organic chemicals

## Abstract

**Simple Summary:**

The study aimed at chemically characterizing the marking fluid of both breeding and non-breeding male cheetahs (*Acinonyx jubatus*). Specifically, it focused on identifying potential differences in pheromones related to sexual behavior/attraction in this species. Furthermore, it aimed at providing more information as a basis for future studies, such as the investigation of specific semiochemicals in the reproductive behavior of cheetahs. The results of this study support the hypothesis of differences in the relative concentration of volatile organic chemicals between male cheetahs; however, they highlight the importance of diet and age on the presence of volatile organic chemicals in the marking fluid.

**Abstract:**

Scent is known to play an important role in the reproduction of cheetahs and other felids. In fact, the presence/odor of a male cheetah has been noted to trigger the estrous cycle in females. The objective of this study was to analyze the marking fluid (MF) of male cheetahs from different breeding groups to determine the composition of volatile organic compounds (VOCs) present, with the aim of identifying potential pheromones relating to sexual behavior/attraction in this species. Four breeding (B; age: 8.9 ± 1.3 years old) and four non-breeding (NB; age: 5.5 ± 0.8 years old) males were selected for this study. Samples were collected into a glass beaker, transferred immediately into a 20 mL glass screw-cap vial with a polytetrafluoroethylene (PTFE) coated silicone septum, and stored until analyzed by headspace solid-phase microextraction (HS-SPME) using gas chromatography–mass spectrometry. A contingency test with Fisher’s exact test, using the frequency (FREQ) procedure of SAS 9.4, was conducted to determine the difference between the number of VOCs identified per breeding group; furthermore, differences in relative concentration (RC) of the identified VOCs between breeding groups were analyzed using ANOVA for repeated measures with the GLIMMIX procedure. From the 13 MF samples analyzed, 53 VOCs were identified, and 12 were identified in all the samples. Five of these (dimethyl disulfide, benzaldehyde, acetophenone, phenol, and indole) are known to be involved in attraction/sexual behavior in mammals. Between the two groups, the RC of indole was significantly higher in the NB group, whereas the RC of dodecanoic acid was significantly higher in the B group. Although not significant, the RC of benzaldehyde was higher in the B versus the NB group. The results of this study do support the hypothesis of differences in VOCs’ between B and NB male cheetahs. However, the overlapping of age and breeding status and the diet differences could not be controlled. Still, the evidence of changes in MF composition in male cheetahs necessitates further studies on possible strategies to improve reproduction in captivity.

## 1. Introduction

Scent deposition has been observed in many species as a tool for communication. In mammals, this occurs mainly by secretions from scent glands on the skin, cheeks, chin, and tail, as well as urine. Regarding scent deposition from their bladder, all felines exhibit the same two behaviors [1,2,3]. Normal urination is one method that involves squatting above the ground before the urine is released [4]. Spraying of urine, termed spray marking behavior, develops around puberty [3,5] and involves the release of one or more streams of urine onto an object [3]. There has been no evidence to prove that other chemical compounds are released into the urine before it is sprayed as a marking fluid [3]. Therefore, it can be assumed that the chemical composition of the urine is the same as that of the marking fluid.

A scent mark forms from the mixture of semiochemicals in the marking fluid, which is left behind on the object to transmit certain messages or signals that are detectable to other animals at a later stage [6,7]. Lipids are fixed to the semiochemicals to increase the persistence time of the scent mark [8]. The definition of semiochemicals is “chemical signal vehicles that carry information between organisms”, and pheromones are a class of semiochemicals that act as chemical signals between individuals of the same species (intraspecific, [9]). The purpose of scent marks is to mark territory, identify neighbors, detect bigger predators and nearby food/prey, signal alarms and attract members of the opposite sex [6,10,11,12,13]. Furthermore, information regarding the age, sex, and reproductive status of an individual can also be obtained from their scent mark [6].

Although several studies have been conducted on the role of semiochemicals in wild and domestic felids [3,5,6,7,10,14,15,16], little is known about their function in cheetahs. In fact, it has been speculated that the lack of interest in cheetah urine among scientists is most likely due to the very little odor it emits [17], which leads to further speculation that the concentration of VOCs potentially related to breeding behavior could be low [17].

A relatively recent study used the voided urine of various male cheetahs to prove the olfactory role of semiochemicals in mate choice by female cheetahs [18], thus proving the involvement of semiochemicals in the urine of male cheetahs in attracting the opposite sex. Furthermore, two studies [11,17] analyzed the volatile organic chemicals (VOCs) in cheetahs’ urine, collected from both male and female cheetahs, with gas chromatography–mass spectrometry (GC–MS) to identify the VOC composition.

Visser (2002) analyzed the urine of four wild male cheetahs located in Namibia, along with three captive males and one captive female located in South Africa. Similarly, Burger et al. (2006) analyzed the urine of two wild males, four captive males, and one captive female located in different facilities in South Africa. Combined, these studies were successful in identifying 53 VOCs, of which 35 were identified in both studies. Despite their success, these studies only compared sexes rather than individuals or specific groups. Therefore, the aim of this study was to chemically characterize the marking fluid (MF) of both breeding and non-breeding males to identify potential differences in pheromones related to sexual behavior/attraction in this species. Such information can be potentially useful to improve reproduction in captivity.

## 2. Materials and Methods

### 2.1. Ethical Approval

This study was approved by the Research Ethics Committee: Animal Care and Use of Stellenbosch University (project number ACU-2019-10425), Stellenbosch, South Africa.

### 2.2. Experimental Location and Animals

Breeding males (B): MF was collected from four B male cheetahs (regularly exposed to females; age: 8.9 ± 1.3 years old) at Feracare Wildlife Centre in Bela-Bela, Gauteng, South Africa (24°40′13.9″ S 28°01′41.8″ E). Their diet at the time of collection consisted of 2 kg chicken mince supplemented with 15 g adult cat dry food (healthy adult, IAMSTM, PROACTIVE HEALTHTM) per day. All B males in this study were housed in separate enclosures from each other, and none of the males were related to each other.

Non-breeding males (NB): MF samples were collected from four NB male cheetahs at Cheetah Outreach in Somerset West, Western Cape, South Africa (34°05′27.1″ S 18°48′48.3″ E). All four males (age: 5.5 ± 0.8 years old) had never had prior breeding experience or exposure to mature females. These animals were housed together with their brothers except during MF collection. Their diet consisted of 2 kg of a mix of chicken, rabbit, turkey, and horse cut in pieces and supplemented with a vitamin and mineral supplement (Predator Powder V-tech, South Africa). Only two of the NB males are related to each other: Kitu and Tobias.

### 2.3. Marking Fluid Collection and Storage

**Figure 1:**
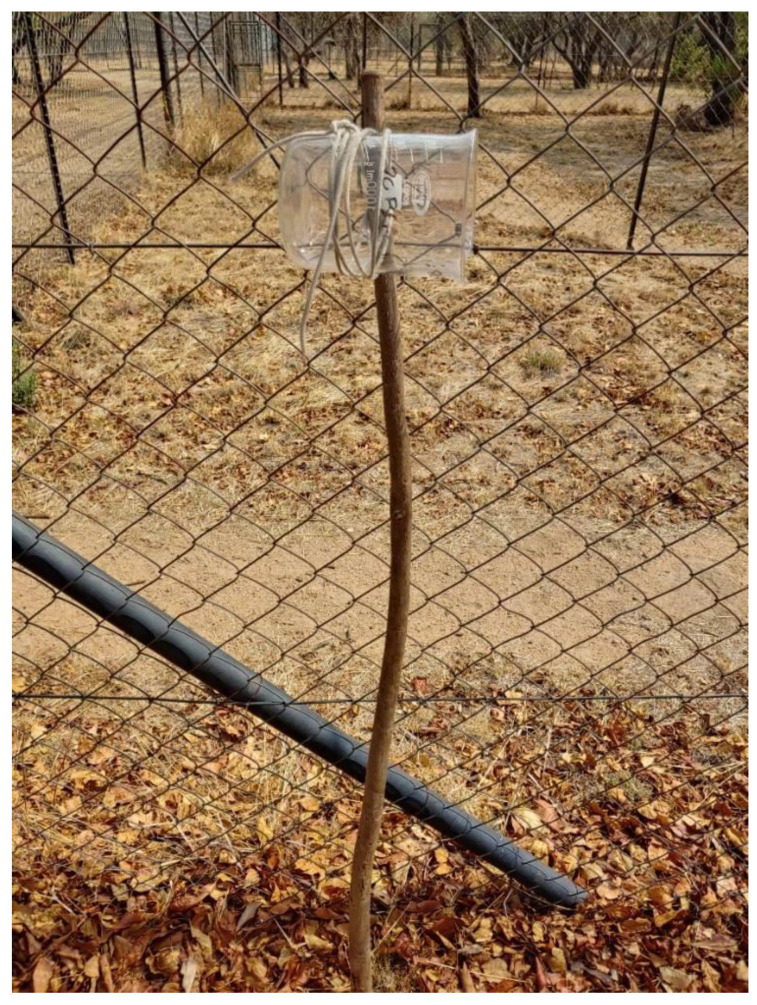
Marking fluid collection device.

The day before sample collection, all glassware was sterilized for 10 min with boiling water and dried in an oven at 150 °C overnight. When a marking event occurred, MF was collected directly into a 600 mL glass beaker (which was either held by hand or tied to a long stick, as shown in Figure 1) and transferred at once into a 20 mL glass screw-cap vial with a polytetrafluoroethylene (PTFE) coated silicone septum.

B males: As per the center’s routine procedure during breeding, males were walked from their own enclosures, either after morning feedings at around 9.00 h or in the afternoon when the weather was cool, and then released in the walkway around the females’ enclosures. Males were then walked back to their enclosures once they were no longer interested in any of the females. Samples were collected during the walk from the male’s first enclosure.

NB males: As per the routine procedure of the facility, the males were separated into their own enclosures before being fed in the morning at around 9.00 h. All samples were collected after the males had finished eating in the morning and begun marking the new enclosure as their new territory.

For each group of animals, all samples were collected within 7 days. A total of 13 samples have been collected, and all of them were stored at −21 °C within an hour after collection before being transported on dry ice and then stored at −80 °C. As proved by [19], a storage temperature of −80 °C is the best temperature for urinary sample stability. Samples stored at this temperature, in fact, show a non-significant reduction in the sum of the VOCs peak areas (when compared to that of the fresh sample) after a period of six months of storage.

### 2.4. Sample Analysis

Headspace solid-phase microextraction (HS-SPME) was performed using 5 mL of MF. To enhance the volatilization of the VOCs to the headspace (HS) and standardize sampling conditions, protonation of the acidic fraction of the MF was accomplished by saturating all the samples with NaCl and adjusting their pH to 3.2 using HCl [20]. Volatile organic compounds (VOCs) were extracted from the HS with a DVB/CAR/PDMS (divinylbenzene/Carboxen/polydimethylsiloxane, 1 cm length, 50/30 µm coating thickness) SPME fiber (Supelco, Bellefonte, PA, USA) at 60 °C for 90 min. Desorption was performed in the GC injector at 240 °C for 5 min. An HP 5890 series II Gas Chromatogram (GC) hyphenated to an Agilent 5973 Mass Spectrometer (MS, Agilent, Palo Alto, CA, USA) was used for GC–MS analysis. The GC injector was operated at 240 °C in splitless mode (2 min). The column used was a DB-FFAP (Agilent) of 30 m length × 0.25 mm i.d. × 0.25 µm film thickness with a constant carrier gas flow of helium at 1 mL/min. The GC oven program was as follows: 40 °C, hold for 2 min, ramped at 5 °C/min to 240 °C, and hold for 2 min. The MS was operated in full-scan mode (scanning from 50–350 amu at a rate of 4.6 scans/s) with ionization energy of 70 eV. The MS transferline, ion source, and quadrupole temperature were held at 240, 230, and 150 °C, respectively.

### 2.5. Identification of VOCs

Data were recorded and processed using Agilent MSD Chemstation (Rev F.01.03) software. VOCs were tentatively identified by comparison of the mass spectrum obtained from individual peaks with those contained in the NIST mass spectral library (NIST v.2.0, US National Institute of Standards and Technology, Gaithersburg, MD, USA). As also performed by other researchers [21,22,23], tentative identification of VOCs was further substantiated by using linear retention indexes (RIs) for identified compounds. The RIs were calculated by using the retention times of linear alkanes by using the following formula:

$$\mathrm{RI}=100[n+\;\frac{\left(tru-trn\right)}{\left(trN-trn\right)}]$$

where ‘*tru*’ is the retention time of the unknown VOC (from the chromatogram), ‘*trn*’ is the retention time of the alkane that elutes before the unknown VOC, ‘*trN*’ is the retention time of the alkane that elutes after the unknown VOC, and ‘*n*’ is the number of carbons of the alkane eluting before the unknown VOC.

Determination of RI was performed in accordance with Van den Dool and Kratz when using linear temperature programming [24]. All calculated RIs per VOC were compared to the RIs from published studies [25] to confirm the presence of the identified VOC. RI differences of less than 10 units between the published data and the calculated RIs were allowed for confirmation of correct identity.

The relative concentration (RC) of each VOC present per sample was calculated from the peak area of a unique ion for the VOC molecule. This analyte peak area was then divided by the peak area of the internal standard (3-octanol), which was added to each sample at a concentration of 50 mg/L before analysis.

### 2.6. Statistical Analysis

A contingency test with Fisher’s exact test, using the frequency (FREQ) procedure of SAS 9.4 (SAS Institute Inc., Cary, NC, USA), was conducted to evaluate possible relationships between the frequency of presence/absence of each VOC and the respective male groups. Furthermore, descriptive analyses of the mean relative concentration of the identified VOCs were conducted using the univariate procedure (SAS 9.4; Cary, NC, USA. SAS Institute Inc). Differences in relative concentration of the identified VOCs between breeding groups were analyzed using analysis of variance (ANOVA) for repeated measures with the generalized linear mixed models (GLIMMIX) procedure. Statistical significance was considered at *p* ≤ 0.05, and tendencies were considered at *p* > 0.05 to *p* ≤ 0.10.

## 3. Results and Discussion

### 3.1. Sample Collection

The initial aim was to collect at least two samples per male for representative individual comparison. However, this greatly depended on how easy the male was to work with; in fact, some males were more difficult to work with, either because they were parent-raised or just because they were more unpredictable in their behavior towards people, thus making it problematic to get close to them. Therefore, for both groups, the number of samples collected per animal ranged from a minimum of one to a maximum of three, with a total of 13 MF samples collected (*n* 5 for the B and *n* 8 for the NB group). All samples were collected from the B males during the walk from the male’s initial enclosure towards the females, with the only exceptions being one sample, which was collected after the male had walked back from the females and to his enclosure, and two more samples, which were collected after the male had entered another male’s enclosure.

### 3.2. Presence of VOCs

A total of 53 VOCs were identified in all the MF samples. Only 13 of them have been previously identified in cheetahs [11,17]. This is most likely due to the animals in the previous studies being housed in various facilities, therefore, receiving different diets/supplementations [26].

Of the 53 identified VOCs, only the following 12 were identified in all the samples: dimethyl disulfide, hexanal, 2-methylmercaptofuran, 3-ethylcyclopentanone, benzaldehyde, acetophenone, 2-furanmethanol, phenol, octanoic acid, nonanoic acid, indole, and dodecanoic acid. Of the 12 commonly identified VOCs, hexanal, 4-heptanone, acetophenone, benzaldehyde, dimethyl disulfide, and phenol have been previously identified in the urine of both male and female cheetahs in other studies [11,17] whereas octanoic acid has only been identified once before in the urine of a female cheetah [11].

A contingency test was conducted to determine the difference between the number of VOCs identified per breeding group: There was no significant difference in the frequency of VOCs identified in the MF of B and NB groups of male cheetahs. Only a tendency (*p* ≤ 0.10) was observed for 3-pentanone, which was observed to be more frequently detected in the NB males (Table 1). This VOC has been identified in the odors released from mating pairs of the insect species *Triatoma infestans*, and it has therefore been associated with communication in terms of sexual behavior in this species [27,28]. However, the role of this VOC has not been clarified in mammals.

### 3.3. Factors Affecting Presence of VOCs

According to a study conducted in 2008 [29], although male cheetahs reach puberty from the age of 3 years old, from a semen quality point of view, many males are suitable for breeding only after the age of 4.5 years, whereas from a behavioral point of view, more mature males result to be more active when introduced to females in estrus; therefore, the optimal breeding age for male cheetahs is from 6 to 11 or 12 years old. Consequently, when working with animals in captivity, such as in the case of this study, reproductive status (breeding or non-breeding) coincides with age group, with the breeding males being older than 6 years (in this study B males were 8.9 ± 1.3 years old) and non-breeding males younger than 6 years (in this study NB males were 5.5 ± 0.8 years old). Furthermore, in South Africa, breeding centers mostly house breeding animals, leaving the largest portion of non-breeding animals to other facilities (e.g.,: sanctuaries, conservation facilities, etc.). This approach adds one more variable to the study; in fact, as reported, the animals involved in this study were located in two different facilities and received different diets, although similar. Thus, although differences have been observed between groups, it cannot be concluded if these are related to the age or the breeding status of the animals or, additionally, to a dietary effect. In fact, it has been observed that the variation regarding the presence/absence of a chemical compound in the MF could be attributed to other factors, such as the endocrine status and environmental influences, such as diet [26].

For example, in a study conducted with mice carrying breast cancer, some VOCs were found in the urine of mice on a high protein diet and not in the urine of any mice on a low protein diet [30]. Therefore, the presence of some VOCs in the MF of only one breeding/age group could be attributed to either the type of protein/s received or the difference in supplementation between the groups (e.g., 3-pentanone), whereas the absence/presence of some VOCs collected from the same cheetah at different times/days could be explained by the endocrine status of the cheetah at the time of collection [26,30]. For example, 2-ethenyl-6-methyl-pyrazine and decanal were identified only in one of the two samples collected from the same animal on different days (15th and 20th of March 2020).

Although it is true that diet has a large effect on the variation of VOCs found in the marking fluid, an animal’s urinary volatile profile also codes for individual identification in chemical communication; therefore, the presence of one VOC in the MF samples collected from only one male could indicate its involvement in individual identification [7,31]. An example from this study would be 2-undecanone from one male and hexadecanal from another male. In the same regard, the presence of one VOC in the MF samples collected from all the males could indicate its involvement in species and/or sexual identification [3]. An example from this study would be the 12 commonly identified VOCs.

### 3.4. Relative Concentrations of the 12 Common VOCs

The mean relative concentration (RC) was calculated only for the 12 VOCs identified in every male cheetah’s MF. This was conducted to identify potential differences regarding the RCs of volatiles between the two breeding groups. Table 2 summarizes the mean RCs and standard deviations of the 12 (common) VOCs in the two groups.

**Figure 2:**
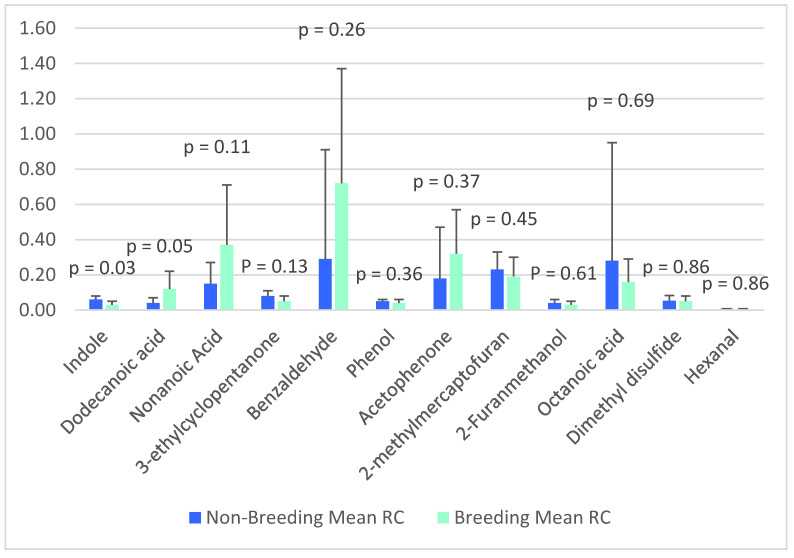
LSM ± SE of the RC of the VOCs identified in the MF in breeding and NB group.

Between the two breeding groups (Figure 2), the RC of indole was significantly higher in the NB group (*p* = 0.03), whereas the RC of dodecanoic acid was significantly higher in the B group (*p* = 0.05).

Indole is known to be involved in sexuality and age differentiation in cats and house mice [7,13,32]. In male domestic cats (Felis *silvestris* catus), this was found to correlate significantly with age resulting in increasing concentrations along with increasing age [33]. In this study, the RC of indole was significantly higher in the younger NB males (*p* = 0.03); therefore, the concentration of indole in the MF could be correlated in a negative manner to age in cheetahs as well. Indole has also been identified in the MF/urine of lions, domestic cats, and tigers [5,7,12,13].

On the other hand, dodecanoic acid is known to be a pheromone that helps regulate oviposition in insects [34]. Although this VOCs involvement in mammalian reproduction is unknown, it has been identified in the urine of both male and female caracals [35], as well as one male Bengal tiger [36]. Therefore, the fact that this VOC is not exclusively identified in female felines means it likely has no function in the birthing process; however, it cannot be excluded from its role in any other function in reproduction in other mammalian species.

Besides indole, the only other four commonly identified VOCs already known as pheromones relating to sexuality in various species are acetophenone (anti-attraction, attraction, responsiveness, stimulates overmarking), benzaldehyde (affects sexual behavior), dimethyl disulfide (attraction, sniffing), and phenol (estrus, sexuality) [7,13,32,37,38].

Phenol was found to be an indication of estrus in female buffaloes, which was hypothesized to be converted to 4-methyl phenol during estrus [39]. In a study measuring the VOC and CO₂ content of human breath, the concentration of phenol and indole rose in some male individuals during sexual arousal, which quickly declined shortly after [40]. Phenol has also been identified in the MF/urine of lions and tigers [7,12,13,36].

Dimethyl disulfide, which has been identified in the urine of male and female snow leopards [41], lions, tigers, and cheetahs [3,7,11,12,13], has also been identified in the vaginal secretions of female hamsters and has been observed to act as a male attractant [13,42]. Like dimethyl disulfide, although acetophenone and benzaldehyde have also been identified in the MF/urine of lions, cheetahs, and tigers [3,7,11,12,13,17,36], their functions in terms of behavior/communication are not known.

Acetophenone and benzaldehyde have been identified as potential ‘sex pheromones’ in female dogs. These VOCs, in fact, increased during the proestrus and estrus stage of the estrous cycle while other foul-smelling sulfureous VOCs decreased, which indicates the attracting effects of the VOCs during proestrus and estrus, as well as the potential repulsive effect of the other sulfureous VOCs during diestrus [43]. Acetophenone excretion is also higher in female wolves than intact males [44], and benzaldehyde is also known to be involved in influencing sexual behavior in lemurs, thereby functioning as a sex pheromone [45].

**Figure 3:**
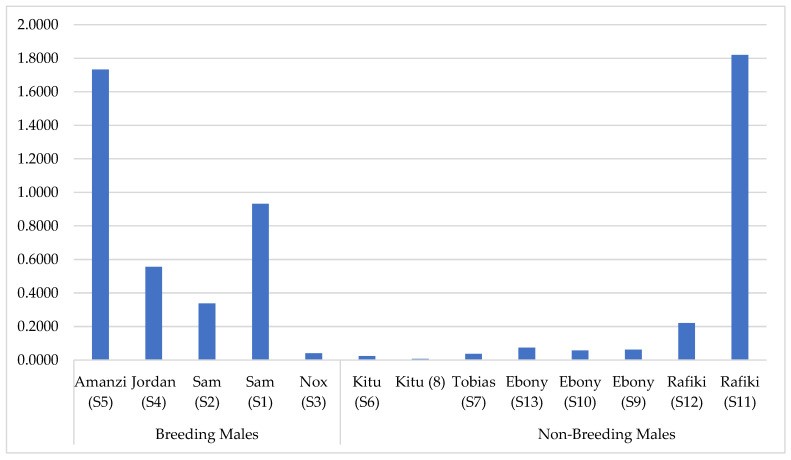
Relative concentration of benzaldehyde per sample of MF collected from breeding and non-breeding male cheetahs.

Although not significantly different, as can be seen in Figure 3, the mean RC of benzaldehyde was numerically higher in the B versus the NB group. Amongst the NB males, the RC of benzaldehyde was high only in one sample (Figure 3). It can be speculated that this high RC of benzaldehyde is because the sample was collected after the male had entered a new enclosure that was previously occupied by another male. In fact, benzaldehyde is also known to be involved in defensive, aggressive, and alarm recruitment behaviors [7,13], so it is quite likely that the new male perceived the previous male as a threat.

The RC of benzaldehyde in the B group was also higher in sample 1 compared to sample 2 (Figure 3). Both samples were collected from the same male, with the first one being collected from the male while around the females and the second one being collected after he returned to his own enclosure. On the other hand, sample 3 was collected from a male that had had no previous exposure to female cheetahs that year. This may indicate the involvement of this specific VOC in sexual/attractive behaviors in cheetahs.

## 4. Conclusions

The aim of this study was to characterize the MF of both B and NB male cheetahs to observe possible differences between the two groups, as well as to identify potential pheromones relating to sexual behavior/attraction in this species.

The results of this study do give an indication as to the differences in RC of VOCs between B and NB male cheetahs. Furthermore, the five common VOCs (dimethyl disulfide, benzaldehyde, acetophenone, phenol, and indole) known to be pheromones involved in attraction/sexual behavior in several species could possibly be involved in similar functions in cheetahs as well. This suggests that the MF of male cheetahs, and specifically certain VOCs within it, may be involved in sexual behavior and, therefore, play a role in mating.

However, since in this study, the age groups (younger and older) corresponded to the non-breeding and breeding group, respectively, it was not possible to distinguish if the differences in RC of the commonly identified VOCs observed from the older B males were related to their exposure to the females or to the physiological differences due to the age, such as sperm quality, or if the two are somehow related. Furthermore, the differences in the diet between the two groups may have affected the absence/presence of certain VOCs [30]. Other limitations, such as the limited sample size, could have affected the results as well.

Further studies controlling diet and age, and involving female cheetahs as well, are indeed necessary to increase data collection on the species itself and to better understand possible differences in RC of VOCs in different breeding groups. Possible strategies could include the use of young breeding males and older non-breeding males for the repetition of this study, as well as an identical diet and supplementation between the two groups. Chemical characterization of the MF/urine collected from female cheetahs in future studies could also help identify differences in VOC composition relating to sex.

Although dimethyl disulfide, benzaldehyde, acetophenone, phenol, and indole have been identified in the urine/MF of other feline species, they are only known to be involved in attractive/reproductive behaviors in other species such as insects, rats, or buffalo [46]. Interestingly, dimethyl disulfide, phenol, and indole are common odorous VOCs associated with fecal matter and livestock manure. In fact, it would be expected to detect these odorants in the scat/urine/waste of wildlife and livestock since the underlying biochemistry to generate these compounds is relatively similar (i.e., digestion of biomass and carcass) and results in similar endpoints of odorous VOCs that could be useful as semiochemicals [12,13,47,48,49]. Therefore, due to these factors, as well as the fact that these VOCs were also identified in every MF sample in this study, further investigation would be necessary to evaluate their possible influence on reproduction in cheetahs.

## Figures and Tables

**Table 1:** VOCs identified in the headspace of MF samples collected from B (Sample 1–5) and NB (Sample 6–13) male cheetahs, including contingency test results.

Functional Group			Sample in Which the VOC Was Identified													***p***
			Breeding Males (8.9 ± 1.3y)					Non-Breeding Males (5.5 ± 0.8y)								
	Name of Identified VOC		1	2	3	4	5	6	7	8	9	10	11	12	13	
**Ketones**	1	3-hexanone			✓			✓	✓	✓						1.00
	2	3-pentanone									✓	✓		✓	✓	0.10
	3	4-heptanone			✓	✓	✓	✓	✓	✓	✓	✓	✓	✓	✓	0.13
	4	3-heptanone		✓	✓	✓		✓	✓	✓	✓		✓	✓	✓	0.51
	5	2-heptanone		✓	✓			✓	✓	✓	✓	✓		✓	✓	0.22
	6	3-octanone	✓	✓	✓	✓	✓	✓	✓	✓	✓	✓	✓		✓	1.00
	7	3-ethylcyclopentanone	✓	✓	✓	✓	✓	✓	✓	✓	✓	✓	✓	✓	✓	0.40
	8	6-methyl-5-hepten-2-one		✓	✓	✓	✓					✓		✓	✓	0.27
	9	3-ethylcyclopent-2-en-1-one		✓												0.38
	10	2-Undecanone									✓	✓			✓	0.49
	11	Benzyl methyl ketone					✓	✓		✓			✓			1.00
	12	1-phenyl-2-propanone		✓	✓											0.13
	13	Acetophenone	✓	✓	✓	✓	✓	✓	✓	✓	✓	✓	✓	✓	✓	1.00
	14	Tetrahydro-6-pentyl-2H-pyran-2-one							✓			✓			✓	0.49
	15	Delta octalactone		✓												0.38
**Aldehydes**	16	Hexanal	✓	✓	✓	✓	✓	✓	✓	✓	✓	✓	✓	✓	✓	1.00
	17	Furfural			✓				✓		✓				✓	1.00
	18	2-furancarboxaldehyde		✓		✓										0.13
	19	Decanal	✓	✓		✓	✓	✓	✓		✓	✓		✓	✓	1.00
	20	Benzaldehyde	✓	✓	✓	✓	✓	✓	✓	✓	✓	✓	✓	✓	✓	1.00
	21	2,6,6-trimethyl-1-cyclohexene-1-carboxaldehyde					✓									0.38
	22	Hexadecanal						✓		✓						0.49
**Esters**	23	Ethyl pentanoate												✓		1.00
	24	Ethyl hexanoate												✓		1.00
	25	Methyl hexadecanoate				✓	✓								✓	0.51
**Alcohols**	26	1-octen-3-ol							✓							1.00
	27	2-ethylhexanol	✓	✓				✓	✓	✓	✓			✓	✓	0.29
	28	2-furanmethanol	✓	✓	✓	✓	✓	✓	✓	✓	✓	✓	✓	✓	✓	1.00
	29	α-methyl benzenemethanol					✓						✓	✓		1.00
	30	Phenylethyl alcohol	✓	✓				✓	✓	✓	✓	✓			✓	0.29
	31	Phenol	✓	✓	✓	✓	✓	✓	✓	✓	✓	✓	✓	✓	✓	1.00
**Acids**	32	Hexanoic acid	✓	✓								✓	✓	✓	✓	1.00
	33	Heptanoic Acid	✓	✓							✓	✓	✓	✓	✓	0.59
	34	Octanoic acid	✓	✓	✓	✓	✓	✓	✓	✓	✓	✓	✓	✓	✓	1.00
	35	Nonanoic Acid	✓	✓	✓	✓	✓	✓	✓	✓	✓	✓	✓	✓	✓	1.00
	36	Decanoic acid	✓	✓	✓	✓	✓		✓		✓	✓	✓	✓	✓	0.49
	37	Undecanoic acid	✓	✓			✓				✓	✓				0.29
	38	Dodecanoic acid	✓	✓	✓	✓	✓	✓	✓	✓	✓	✓	✓	✓	✓	1.00
	39	Hexadecanoic acid					✓						✓	✓		1.00
**Hydrocarbons**	40	Styrene					✓						✓			1.00
	41	Octadecane					✓									0.38
	42	2-methylnaphthalene					✓									0.38
**Sulfur containing compounds**	43	Dimethyl disulfide	✓	✓	✓	✓	✓	✓	✓	✓	✓	✓	✓	✓	✓	1.00
	44	2-methylmercaptofuran	✓	✓	✓	✓	✓	✓	✓	✓	✓	✓	✓	✓	✓	1.00
	45	Dimethyl trisulfide	✓	✓	✓		✓	✓	✓	✓	✓		✓	✓	✓	1.00
**Nitrogen-Containing Compounds**	46	1-methyl-1H-pyrrole							✓							1.00
	47	2-ethenyl-6-methyl-pyrazine						✓								1.00
	48	Ethyl carbamate			✓	✓	✓					✓		✓	✓	0.59
	49	2-piperidinone			✓	✓	✓						✓	✓	✓	0.59
	50	Indole	✓	✓	✓	✓	✓	✓	✓	✓	✓	✓	✓	✓	✓	1.00
**Terpenoids**	51	Linalool							✓							1.00
	52	α-terpineol									✓	✓			✓	0.49
**Furans**	53	2-pentyl-furan			✓				✓				✓	✓	✓	0.56

**Table 2:** Mean relative concentration, estimated concentration, and standard deviation of the 12 common VOCs per breeding group.

VOC	RC (Unitless)	
	Non-Breeding (5.5 ± 0.8y)	Breeding (8.9 ± 1.3y)
**Dimethyl disulfide**	0.05 ± 0.03	0.05 ± 0.03
**Hexanal**	0.01 ± 0.00	0.01 ± 0.00
**2-methylmercaptofuran**	0.23 ± 0.10	0.19 ± 0.11
**3-ethylcyclopentanone**	0.08 ± 0.03	0.05 ± 0.03
**Benzaldehyde**	0.29 ± 0.62	0.72 ± 0.65
**Acetophenone**	0.18 ± 0.29	0.32 ± 0.25
**2-Furanmethanol**	0.04 ± 0.02	0.03 ± 0.02
**Phenol**	0.05 ± 0.01	0.04 ± 0.02
**Octanoic acid**	0.28 ± 0.67	0.16 ± 0.13
**Nonanoic acid**	0.15 ± 0.12	0.37 ± 0.34
**Indole**	0.06 ± 0.02	0.03 ± 0.02
**Dodecanoic acid**	0.04 ± 0.03	0.12 ± 0.1

## Data Availability

Raw data will be made available upon request to the corresponding author.
